# Rho-Kinase/ROCK Phosphorylates PSD-93 Downstream of NMDARs to Orchestrate Synaptic Plasticity

**DOI:** 10.3390/ijms24010404

**Published:** 2022-12-26

**Authors:** Emran Hossen, Yasuhiro Funahashi, Md. Omar Faruk, Rijwan Uddin Ahammad, Mutsuki Amano, Kiyofumi Yamada, Kozo Kaibuchi

**Affiliations:** 1 Department of Cell Pharmacology, Graduate School of Medicine, Nagoya University, 65 Tsurumai, Nagoya 466-8550, Japan; 2 Division of Cell Biology, International Center for Brain Science, Fujita Health University, Toyoake 470-1192, Japan; 3 Department of Biochemistry and Molecular Biology, Faculty of Biological Sciences, University of Dhaka, Dhaka 1000, Bangladesh; 4 Department of Cellular and Molecular Medicine, University of California San Diego, La Jolla, CA 92093, USA; 5 Department of Neuropsychopharmacology and Hospital Pharmacy, Graduate School of Medicine, Nagoya University, 65 Tsurumai, Nagoya 466-8550, Japan

**Keywords:** PSD-93, Rho-kinase, phosphorylation, dendritic spine, LTP

## Abstract

The N-methyl-D-aspartate receptor (NMDAR)-mediated structural plasticity of dendritic spines plays an important role in synaptic transmission in the brain during learning and memory formation. The Rho family of small GTPase RhoA and its downstream effector Rho-kinase/ROCK are considered as one of the major regulators of synaptic plasticity and dendritic spine formation, including long-term potentiation (LTP). However, the mechanism by which Rho-kinase regulates synaptic plasticity is not yet fully understood. Here, we found that Rho-kinase directly phosphorylated discs large MAGUK scaffold protein 2 (DLG2/PSD-93), a major postsynaptic scaffold protein that connects postsynaptic proteins with NMDARs; an ionotropic glutamate receptor, which plays a critical role in synaptic plasticity. Stimulation of striatal slices with an NMDAR agonist induced Rho-kinase-mediated phosphorylation of PSD-93 at Thr612. We also identified PSD-93-interacting proteins, including DLG4 (PSD-95), NMDARs, synaptic Ras GTPase-activating protein 1 (SynGAP1), ADAM metallopeptidase domain 22 (ADAM22), and leucine-rich glioma-inactivated 1 (LGI1), by liquid chromatography-tandem mass spectrometry (LC-MS/MS). Among them, Rho-kinase increased the binding of PSD-93 to PSD-95 and NMDARs. Furthermore, we found that chemical-LTP induced by glycine, which activates NMDARs, increased PSD-93 phosphorylation at Thr612, spine size, and PSD-93 colocalization with PSD-95, while these events were blocked by pretreatment with a Rho-kinase inhibitor. These results indicate that Rho-kinase phosphorylates PSD-93 downstream of NMDARs, and suggest that Rho-kinase mediated phosphorylation of PSD-93 increases the association with PSD-95 and NMDARs to regulate structural synaptic plasticity.

## 1. Introduction

Synaptic plasticity is a key mechanism of learning and memory. During synaptic plasticity, many changes occur between neurons, such as changes in presynaptic vesicles, Ca^2+^ concentrations, and neurotransmitter receptors. These alterations regulate the dendritic spine volume, which is increased during long-term potentiation (LTP) and decreased during long-term depression (LTD) [1,2]. Moreover, the enlargement of dendritic spine volume in LTP is crucial for strengthening connections between neurons which is essential for synaptic transmission [3,4]. During LTP, the activation of RhoA regulates the cytoskeleton, which subsequently enlarges the spine volume by increasing actin polymerization and incorporating α-amino-3-hydroxy-5-methyl-4-isoxazolepropionic acid receptors (AMPARs) in the postsynaptic region [5,6]. One study using fluorescence lifetime imaging after two-photon glutamate uncaging has shown that RhoA affects transient and sustainable changes in dendritic spine volume [7]. RhoA is activated by glutamate, which is one of the most abundant neurotransmitters in excitatory synapses. Glutamate-containing presynaptic vesicles release glutamate into the synaptic cleft, which subsequently binds to postsynaptic glutamate receptors, such as AMPARs, N-methyl-D-aspartate receptors (NMDARs), and kainate receptors [8]. Among these glutamate receptors, glutamate binds to AMPARs and increases Na^+^ influx via their pores upon glutamate binding. This Na^+^ entry causes depolarization of postsynaptic neurons, which subsequently removes Mg^2+^ from NMDARs that blocks Ca^2+^ influx upon glutamate stimulation. By removing Mg^2+^, glutamate neurotransmitters initiate Ca^2+^ influx through the NMDARs, thereby activating CaMKII via autophosphorylation [9,10]. Active CaMKII activates the RhoA–Rho-kinase cascade, which induces dendritic spine enlargement [6,7,11]. However, the mechanism by which Rho-kinase regulates dendritic spine formation during LTP is not yet fully understood.

To identify in vivo Rho-kinase candidate substrates, we previously reported a novel phosphoproteomic method that uses affinity beads coated with 14-3-3 bait proteins to capture phospho-Ser/Thr containing peptides [12]. HeLa cells were stimulated with calyculin A (a phosphatase inhibitor) with/without Y-27632 (a Rho-kinase inhibitor). Treatment with calyculin A increased the phosphorylation of several proteins, including Rho-kinase substrates. However, the pretreatment with Y-27632 specifically inhibited the phosphorylation by Rho-kinase. A comparison of calyculin A treatment combined with calyculin A and Y-27632 treatment identified more than 100 Rho-kinase candidate substrates, including well-known substrates such as protein phosphatase 1 regulatory subunit 12A (PPP1R12A/MYPT1) [12]. The data obtained from phosphoproteomics were deposited in our online database, the Kinase-Associated Neural Phospho-Signaling database (KANPHOS; https://kanphos.neuroinf.jp, accessed on 10 December 2021) [13]. Similar data were obtained for neurons in the striatum. We hypothesized that the KANPHOS data included Rho-kinase candidate substrates that were involved in synaptic plasticity. We identified discs large MAGUK scaffold protein 2 (DLG2/PSD-93) as a Rho-kinase candidate substrate in the striatum. PSD-93 is one of the major postsynaptic scaffold proteins and connects NMDARs to postsynaptic proteins. PSD-93 forms a heterodimer with DLG4 (PSD-95) and plays an important role in synaptic plasticity [14].

In the present study, we examined whether Rho-kinase phosphorylates PSD-93 to further elucidate the function of this kinase in synaptic plasticity. We found that Rho-kinase phosphorylates PSD-93 downstream of NMDARs and that Rho-kinase increases the interactions of PSD-93 with PSD-95 but decreases the interaction of PSD-93 with synaptic Ras GTPase-activating protein 1 (SynGAP1), ADAM metallopeptidase domain 22 (ADAM22), and leucine rich glioma inactivated 1 (LGI1). In conclusion, our observations improve the current understanding of how Rho-kinase regulates the interaction of synaptic proteins with NMDARs and AMPARs to orchestrate synaptic plasticity.

## 2. Results

### 2.1. Rho-Kinase Phosphorylates PSD-93 In Vitro

**Figure 1:**
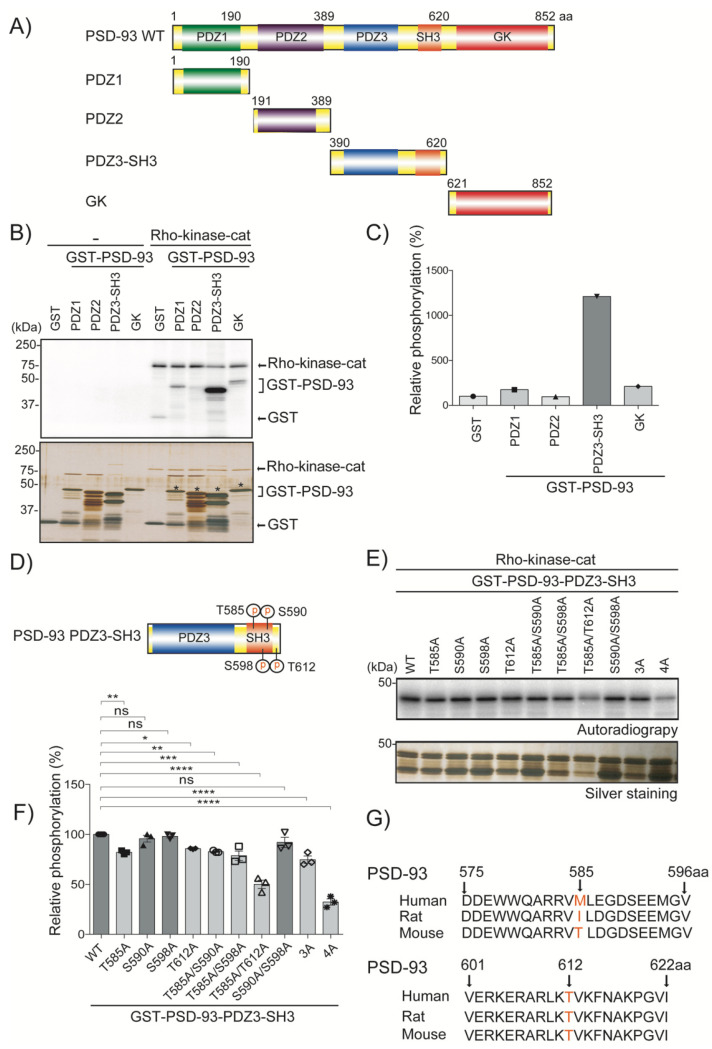
Rho-kinase phosphorylates PSD-93 in vitro. (**A**) PSD-93 domain organization: three PDZ domains (PDZ1 PDZ2 and PDZ3), one SH3 domain and one GK domain. The numbers represent amino acids. (**B**) Constitutively active Rho-kinase phosphorylated PSD-93 at the PDZ3-SH3 domain. Purified GST and GST-PSD-93 domain proteins were incubated with [γ^32^P] ATP in the presence or absence of Rho-kinase-cat in vitro. Samples were subjected to SDS-PAGE and silver staining followed by autoradiography. Asterisks denote intake GST-PSD-93 fusion domain proteins. The arrows denote recombinant Rho-kinase-cat and GST. Third bracket represents PSD-93 domain proteins. (**C**) The bar diagram shows the relative phosphorylation (%) of PSD-93 domain proteins. (**D**) Schematic presentation of PSD-93 phosphorylation sites. The PSD-93-PDZ3-SH3 domain contains four putative phosphorylation sites based on Rho-kinase consensus motifs of R/KXXpS/T and R/KXpS/T (R, arginine; K, lysine; X, any amino acid; S, serine and T, threonine). P denotes phosphate group. (**E**) Rho-kinase phosphorylated PSD-93-PDZ3-SH3 domain at four different sites. Purified wild-type and phospho-deficient mutants of PSD-93-PDZ3-SH3 were incubated with Rho-kinase-cat along with [γ^32^P] ATP in vitro. Next, the SDS-boiled samples were subjected to SDS-PAGE and silver staining followed by autoradiography. The upper panel shows autoradiography, and the lower panel shows a silver staining image. (**F**) Bar diagram showing the relative phosphorylation (%) of PSD-93 domain proteins. The horizontal lines indicate the mean ± SEM of three independent experiments.*, **, *** and **** represent *p* < 0.05, *p* < 0.01, *p* < 0.001 and *p* < 0.0001, respectively, for Dunnett’s multiple comparisons test. “ns” denotes “not significant”. (**G**) The amino acid sequence homology of PSD-93 phosphorylation sites (Thr585 and Thr612) in different species (humans, rats, and mice) is shown in a schematic graph. The numbers represent the amino acid position.

PSD-93 is composed of three postsynaptic density 95/discs large/zona occludens-1 (PDZ) domains, a Src homology 3 (SH3) domain, and an enzymatically inactive guanylate kinase-like (GK) domain (Figure 1A). To examine whether Rho-kinase directly phosphorylates PSD-93, an in vitro phosphorylation assay was performed using four fragments of PSD-93: PDZ1 (amino acids 1–190), PDZ2 (amino acids 191–389), PDZ3-SH3 (amino acids 390–620), and GK (amino acids 621–852). Purified GST-PSD-93 fragments were incubated with [γ32P] ATP in vitro in the presence or absence of Rho-kinase-catalytic domain (Rho-kinase-cat), which acts as constitutively active form of Rho-kinase [15], and this activity is abolished by the specific Rho-kinase inhibitor in vitro [16,17]. Rho-kinase-cat phosphorylated PDZ3-SH3, but not PDZ1, PDZ2 or GK (Figure 1B,C), indicating that Rho-kinase directly phosphorylates PDZ-SH3 of PSD-93. To identify the sites of PDZ3-SH3 phosphorylation by Rho-kinase, we constructed alanine mutants of the Ser/Thr sites of PDZ3-SH3 and performed an in vitro phosphorylation assay. A single alanine substitution at Thr585 and Thr612 reduced the phosphorylation by Rho-kinase-cat by approximately 20% (*p* < 0.01 and *p* < 0.05, respectively), while substitution at Ser590 and Ser598 did not alter the phosphorylation levels (Figure 1D–F). The double mutation 2A (T585A/T612A) reduced the phosphorylation by approximately 50% (*p* < 0.0001), the triple mutation 3A (T585A/S590A/S598A) reduced the phosphorylation by approximately 25% (*p* < 0.0001), and the quadruple mutant 4A (T585A/S590A/S598A/T612A) reduced the phosphorylation by approximately 70% (*p* < 0.0001) (Figure 1D–F), suggesting that Thr585 and Thr612 are the major phosphorylation sites, whereas Ser590 and Ser598 are the minor phosphorylation sites of Rho-kinase. Notably, the sequence homology of the Thr612 PSD-93 phosphorylation site is well conserved in mammals (humans, rats, and mice), whereas the Thr585 PSD-93 phosphorylation site is specific to mice. The amino acids sequence was taken from a protein database (https://www.uniprot.org, accessed on 10 August 2021) (Figure 1G).

### 2.2. Rho-Kinase Phosphorylates PSD-93 at Thr612 in Striatal Slices

To monitor the phosphorylation of PSD-93 by Rho-kinase, we generated an antibody that specifically recognized PSD-93 phosphorylation at Thr612. The sensitivity and specificity of this antibody were examined using immunoblotting analysis. In this analysis, in vitro phosphorylation of GST-PSD-93-PDZ3-SH3 by Rho-kinase was identified by using the anti-pT612 PSD-93 phospho-antibody in a dose-dependent manner (Supplementary Materials, Figure S1A,B). The phospho-specific antibody detected phosphorylated GST-PSD-93-PDZ3-SH3 by Rho-kinase without cross-reacting with the nonphosphorylated form of GST-PSD-93-PDZ3-SH3 (*p* < 0.0001) (Supplementary Materials, Figure S1A,B). Furthermore, the specificity of the antibody was examined using in vitro phosphorylation assays with phospho-deficient PSD-93 mutants. The pT612 PSD-93 antibody recognized the wild-type and the T585A, S590A, and S598A mutants, but did not recognize the T612A and 4A GST-PSD-93-PDZ3-SH3 mutants (*p* < 0.0001 and *p* < 0.0001, respectively) (Supplementary Materials, Figure S1C,D). This result indicates that the anti-pT612 PSD-93 antibody specifically recognizes PSD-93 phosphorylation at Thr612.

**Figure 2:**
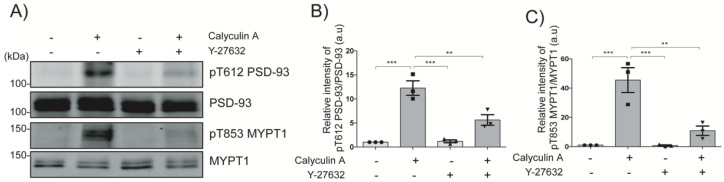
Rho-kinase phosphorylates PSD-93 at Thr612 in striatal slices. (**A**) Striatal slices were incubated with calyculin A (250 nM) for 60 min and/or Y-27632 (20 μM) for 60 min. The samples were analyzed by immunoblot analysis using anti-pT612 PSD-93, anti-PSD-93, anti-pT853 MYPT1, and anti-MYPT1 antibodies. (**B**,**C**) The bar diagram shows the quantification of the immunoblot data for pT612 PSD-93 and pT853 MYPT1, respectively. The horizontal lines indicate the mean ± SEM of three independent experiments. ** and *** represent *p* < 0.01 and *p* < 0.001, respectively, for Tukey’s multiple comparisons test.

To assess whether Rho-kinase phosphorylates PSD-93 at Thr612 ex vivo, striatal slices were cultured and stimulated with calyculin A (a phosphatase inhibitor) and/or Y-27632 (a specific Rho-kinase inhibitor). Calyculin A treatment increased PSD-93 at Thr612 phosphorylation approximately 12-fold (*p* < 0.001) and MYPT1 at Thr853 phosphorylation approximately 50-fold (*p* < 0.001), which is a specific Rho-kinase substrate, at Thr853 [18] (Figure 2A–C). Pretreatment with Y-27632 significantly inhibited calyculin A-induced phosphorylation of PSD-93 at Thr612 (*p* < 0.01) and MYPT1 at Thr85 (*p* < 0.01) (Figure 2A–C). These results indicate that Rho-kinase phosphorylates PSD-93 at Thr612 in striatal slices.

### 2.3. NMDA Induces the Phosphorylation of PSD-93 at Thr612 in Striatal Slices

**Figure 3:**
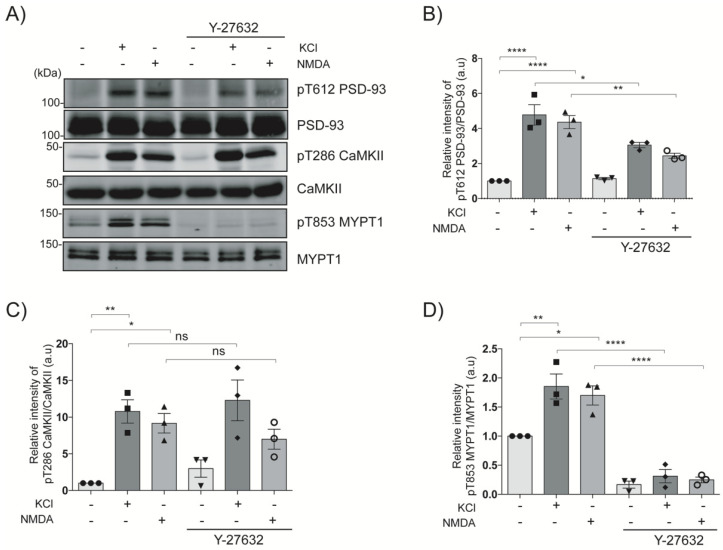
Rho-kinase phosphorylates PSD-93 at Thr612 in striatal slices downstream of NMDARs. (**A**) Striatal slices were treated with or without the Rho-kinase inhibitor Y-27632 (20 μM for 60 min) and then treated with DMSO, or high K^+^ (KCl, 40 mM) for 15 sec, or NMDA (100 μM) for 15 s. The samples were analyzed with immunoblot analysis with anti-pT612 PSD-93, anti-PSD-93, anti-pT286 CaMKII, anti-CaMKII, anti-pT853 MYPT1, and anti-MYPT1 antibodies. (**B**–**D**) The bar diagram shows the quantification of the immunoblot data for pT612 PSD-93, pT286 CaMKII, and pT853 MYPT1, respectively. The horizontal lines indicate the mean ± SEM of three independent experiments. *, ** and **** represent, *p* < 0.05, *p* < 0.01, *p* < 0.0001, respectively, and “ns” denotes “not significant”, for Tukey’s multiple comparisons test.

The NMDARs stimulation induces the influx of Ca^2+^ through NMDARs and activates CaMKII, which then activates the RhoA–Rho-kinase pathway [7,11]. The NMDA (an NMDAR agonist) and high K^+^ induced the CamKII autophosphorylation at Thr286 downstream of NMDARs [19,20]. To evaluate whether Rho-kinase phosphorylates PSD-93 downstream of NMDARs, striatal slices were treated with high K^+^ or NMDA to induce Ca^2+^ influx through NMDARs. Respective treatment of striatal slices with high K^+^ and NMDA induced the phosphorylation of CaMKII at Thr286 approximately 12-fold (*p* < 0.01) and 10-fold (*p* < 0.05), respectively; that of MYPT1 at Thr853, approximately 2-fold (*p* < 0.01) and 1.5-fold (*p* < 0.05), respectively; and of PSD-93 at Thr612 approximately 5-fold (*p* < 0.0001) and 4-fold (*p* < 0.0001), respectively (Figure 3A–D). Pretreatment with Y-27632 significantly inhibited the high K^+^- or NMDA-induced phosphorylation of MYPT1 (*p* < 0.0001 and *p* < 0.0001, respectively) and of PSD-93 (*p* < 0.05 and *p* < 0.01, respectively), but not that of CaMKII (Figure 3A–D). Moreover, pre-treatment with MK-801 (NMDAR antagonist) significantly inhibited the high K+ or NMDA-induced phosphorylation of CaMKII (*p* < 0.01 and *p* < 0.001, respectively), of MYPT1 (*p* < 0.05 and *p* < 0.05, respectively), and of PSD-93 (*p* < 0.01 and *p* < 0.05, respectively) (Supplementary Materials, Figure S2A–D). These results indicate that Rho-kinase phosphorylates PSD-93 at Thr612 and MYPT1 at Th853 downstream of NMDARs in striatal slices under conditions of NMDAR agonist-mediated CaMKII stimulation.

### 2.4. Identification of PSD-93-Binding Proteins by Liquid Chromatography-Tandem Mass Spectrometry (LC-MS/MS) Analysis

**Figure 4:**
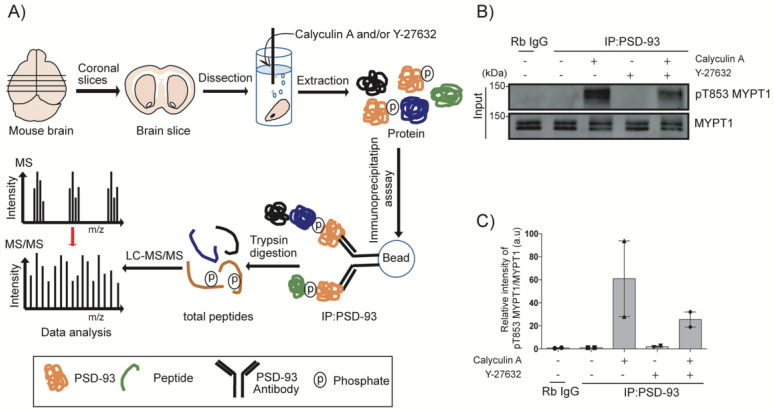
Identification of PSD-93-binding proteins by LC-MS/MS. (**A**) Schematic presentation of the LC-MS/MS method using striatal slices. Coronal striatal slices were taken from mice brain with vibratome and treated with calyculin A (250 nM) for 60 min and/or Y-27632 (20 µM) for 60 min—and then subjected to immunoprecipitation using an anti-PSD-93 antibody. The Trypsin/Lys-C was used to digest immunoprecipitated proteins, which were then subjected to LC-MS/MS to identify PSD-93 interacting proteins. (**B**) The input lysate from striatal slices was analyzed by immunoblot analysis with anti-pT853 MYPT1 and anti-MYPT1 antibodies. (**C**) The bar diagrams show the quantitative analysis results of immunoblotting.

PSD-93 is an adaptor protein that can interact with receptors on the postsynaptic membrane, such as NMDARs and AMPARs, contributing to receptor stabilization [21]. To investigate the effect of PSD-93 phosphorylation by Rho-kinase on its interactions with synaptic proteins and receptors, we performed an immunoprecipitation assay using calyculin A- and/or Y-27632-stimulated striatal slices followed by LC-MS/MS analysis (Figure 4A). We confirmed MYPT1 phosphorylation by immunoblotting after the stimulation of striatal slices with calyculin A and/or Y-27632. Calyculin A increased the phosphorylation of MYPT1 at Thr853 approximately 60-fold, which was inhibited approximately 40-fold by Y-27632 (Figure 4B,C). We categorized LC-MS/MS data based on two criteria: first, the ion intensity of the sample-control had to be more than 10 times higher than that of the IgG-control; and second, the ion intensity of the calyculin A-treated sample had to be lower or higher than that of the sample-control as well as the Y-27632-treated sample. We identified 28 proteins as PSD-93-binding proteins. We further classified these 28 proteins into two categories. The first category contained 12 phosphorylation-dependent positively regulated proteins (whose binding was increased by calyculin A and repressed by Y-27632), namely Agap3, Dlg1, Dlg4, Dlgap1, Dlgap4, Grin1, Grin2a, Grin2b, Griks, Iqsec2, Nrcam and Rtn3 (Table 1). The second contained 16 phosphorylation-dependent negatively regulated proteins (whose binding was reduced by calyculin A and restored by Y-27632), namely ADAM22, Begain, Cacna2d1, Cit, Cnksr2, Dclk1, Dlgap2, Dlgap3, Kcnj4, LGI1, Lrrc7, Map4, Prph, Shisa7, Shank3 and SynGAP1 (Table 2).

### 2.5. Rho-Kinase Regulates the Interaction of PSD-93 with Partner Proteins

**Figure 5:**
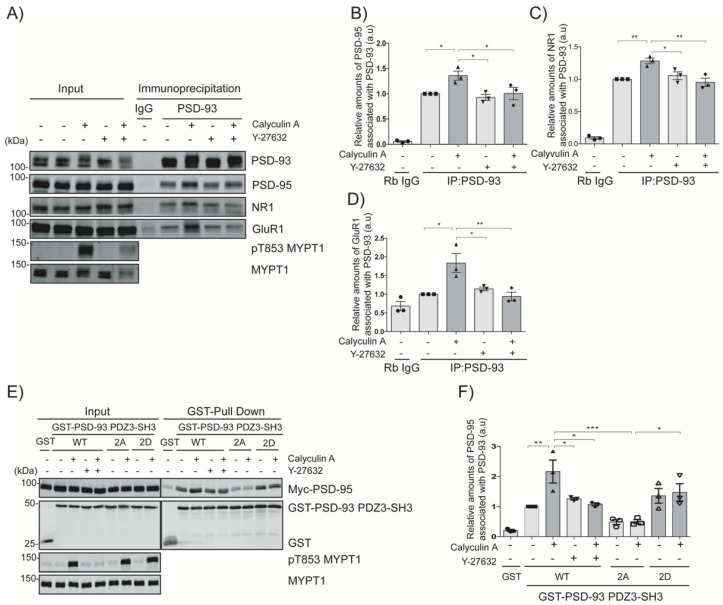
Rho-kinase positively regulates the interaction of PSD-93 with PSD-95, NMDARs and AMPARs. (**A**) PSD-93 phosphorylation by Rho-kinase increases its interaction with PSD-95, NR1 and GluR1 in striatal slices. Striatal slices were treated with calyculin A (250 nM) for 60 min after they were pretreated with Y-27632 (20 µM) for 60 min and then immunoprecipitated using an anti-PSD-93 antibody. The precipitated proteins were subjected to immunoblot analysis with antibodies against PSD-93, PSD-95, NR1, GluR1, pT853 MYPT1, and MYPT1 antibodies. (**B**–**D**) The bar diagram shows the statistical analysis results for the immunoblot data. The horizontal lines indicate the mean ± SEM of three independent experiments. * and ** represent *p* < 0.05 and *p* < 0.01, respectively, for Tukey’s multiple comparisons test. (**E**) Rho-kinase increased the interaction of PSD-93 with PSD-95 in COS7 cells: COS7 cells were cotransfected with Myc-PSD-95 and GST-PSD-93-PDZ3-SH3 (WT, phosphodeficient and phosphomimetic mutants) and then treated with calyculin A (50 nM) for 12 min with or without pretreatment with Y-27632 (20 µM). The samples were subjected to a GST pull-down assay. (**F**) The bar diagram shows the quantification of the immunoblot data. The horizontal lines indicate the mean ± SEM of three independent experiments. *, ** and *** represent *p <* 0.05, *p <* 0.01 and *p <* 0.001, respectively, for Tukey’s multiple comparisons test.

From the LC-MS/MS data, we identified Dlg4 (also known as PSD95), Grin1 (also known as NR1, an NMDAR1 subunit), Grin2a (also known as NR2A, an NMDAR2A subunit), Grin2b (also known as NR2B, an NMDAR2B subunit) as the phosphorylation-dependent positively regulated PSD-93 binding partners (Table 1). To examine whether Rho-kinase positively regulates the interaction of PSD-93 with putative binding proteins, such as PSD-95 and NMDARs, mouse striatal slices were treated with calyculin A and/or Y-27632, and an immunoprecipitation assay using an anti-PSD-93 antibody was performed. When PSD-93 was immunoprecipitated from striatal lysate, PSD-95, NR1, and GluR1 (a subunit of AMPARs) were coprecipitated (Figure 5A–D). The coprecipitation of PSD-93 with PSD-95 (approximately 1.5-fold, *p* < 0.05), NR1 (approximately 1.2-fold, *p* < 0.01), and GluR1 (approximately 2-fold, *p* < 0.05) was increased by treatment with calyculin A, whereas pretreatment with Y-27632 significantly inhibited these interactions (*p* < 0.05, *p* < 0.01 and *p* < 0.01, respectively) (Figure 5A–D). These results suggest that Rho-kinase positively regulates the interaction of PSD-93 with PSD-95, NMDARs and AMPARs. To further explore the relationship between PSD-93 and PSD-95, a robust and easily transfectable cell line of COS7 cells was cotransfected with wild-type PSD-93 (PSD-93-PDZ3-SH3-WT), phospho-deficient PSD-93 (PSD-93-PDZ3-SH3-T585A/T612A), or phospho-mimic mutant PSD-93 (PSD-93-PDZ3-SH3-T585D/T612D) along with wild type Myc-PSD-95 and then stimulated with calyculin A and/or Y-27632. Treatment of COS7 cells with calyculin A increased the interaction of wild-type PSD-93-PDZ3-SH3-WT with Myc-PSD-95 compared to the control 2.5-fold (*p* < 0.01), while pretreatment with Y-27632 significantly inhibited this interaction (*p* < 0.05) (Figure 5E,F). The phospho-deficient mutant (PSD-93-PDZ3-SH3-T585A/T612A) showed a reduced interaction with Myc-PSD-95 (*p* < 0.001), and the phospho-mimetic mutant (PSD-93-PDZ3-SH3-T585D/T612D) showed an enhanced interaction with Myc-PSD-95 (*p* < 0.05) (Figure 5E,F). These results indicate that Rho-kinase phosphorylates PSD-93, thereby positively regulating the interaction of PSD-93 with PSD-95.

From the LC-MS/MS data, we found that PSD-93 interacts with SynGAP1, ADAM22 and LGI1 (Table 2). Moreover, a recent study has shown that the dissociation of SynGAP1 from PSD-95 upon NMDAR-mediated activation reduces the dendritic spine volume and synaptic plasticity in neurons [11]. Another study has revealed that the ADAM22-LGI1-PSD-95 interaction regulates synaptic transmission through AMPA and NMDA receptors in neurons [22]. Since the PSD-93 is closely related to PSD-95 in terms of expression, amino acid sequence, domain organization and functions [23], we hypothesized that the SynGAP1, ADAM22 and LGI1 have similar kind of interactions with PSD-93. We therefore examined whether Rho-kinase negatively regulates the interaction of PSD-93 with SynGAP1, ADAM22, and LGI1 or not. When PSD-93 was immunoprecipitated from striatal lysate, SynGAP1, ADAM22, and LGI1 were also coprecipitated (Supplementary Materials, Figure S3A–D). The coprecipitation of PSD-93 with SynGAP1 (*p* < 0.001), ADAM22 (*p* < 0.01), and LGI1 (*p* < 0.001) was decreased by approximately half on treatment with calyculin A, whereas pretreatment with Y-27632 significantly abolished this effect (*p* < 0.001, *p* < 0.001 and *p* < 0.001, respectively) (Supplementary Materials, Figure S3A–D). These results demonstrate that Rho-kinase negatively regulates the interaction of PSD-93 with SynGAP1, ADAM22, and LGI1. We further explored the relationship between PSD-93 and SynGAP1: COS7 cells were cotransfected with wild-type PSD-93 (PSD-93-PDZ3-SH3-WT), phospho-deficient PSD-93 (PSD-93-PDZ3-SH3-T585A/T612A), or phospho-mimic mutant PSD-93 (PSD-93-PDZ3-SH3-T585D/T612D) along with wild type Myc-SynGAP1 and then stimulated with calyculin A and/or Y-27632. Treatment of COS7 cells with calyculin A significantly decreased the interaction (*p* < 0.0001) between wild-type PSD-93-PDZ3-SH3-WT and SynGAP1. Pretreatment with Y-27632 partially alleviated this effect (*p* < 0.05) (Supplementary Materials, Figure S3E,F). The phospho-deficient mutant (PSD-93-PDZ3-SH3-T585A/T612A) interacted with SynGAP1 to an extent similar to that of PSD-93-PDZ3-SH3-WT (*p* < 0.0001), and this interaction was not affected by treatment with calyculin A (*p* < 0.0001) (Supplementary Materials, Figure S3E,F). The phospho-mimetic mutant (PSD-93-PDZ3-SH3-T585D/T612D) showed a significantly reduced interaction with SynGAP1 (*p* < 0.0001) (Supplementary Materials, Figure S3E,F). These results reveal that Rho-kinase phosphorylates PSD-93 and negatively regulates the interaction of PSD-93 with SynGAP1.

### 2.6. Chemically Induced-LTP Increases the Rho-Kinase-Mediated Phosphorylation of PSD-93 and the Colocalization of PSD-93 with PSD-95

**Figure 6:**
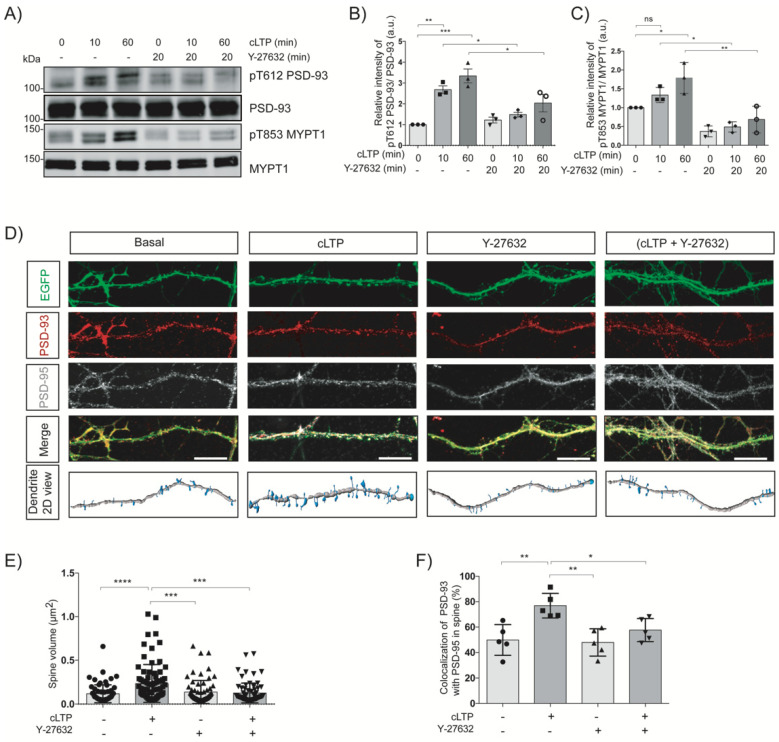
Chemically induced-LTP increases the Rho-kinase-mediated phosphorylation of PSD-93 and the colocalization of PSD-93 with PSD-95. (**A**) Chemical-LTP induces the PSD-93 phosphorylation in cultured striatal neurons. Striatal neurons were cultured until DIV21 and then neurons were treated with glycine (200 µM) after pretreatment with the Rho-kinase inhibitor Y-27632 (20 µM). The samples were analyzed by immunoblot analysis of anti-pT612 PSD-93, anti-PSD-93, anti-pT853 MYPT1, and anti-MYPT1 antibodies. (**B**,**C**) The horizontal lines represent the mean ± SEM of three independent experiments. *, ** and *** represent *p <* 0.05, *p <* 0.01, *p <* 0.001, respectively, and “ns” denotes “not significant”, for Tukey’s multiple comparisons test. (**D**) Colocalization of PSD-93 with PSD-95 during chemical-LTP in cultured primary striatal neurons. The neurons were cultured until DIV14 and infected with AAV-EGFP and AAV-Cre virus. After DIV21, the neurons were treated with glycine to induce chemical-LTP and the immunostaining was performed with anti-GFP (green), anti-PSD-93 (red) and anti-PSD-95 (white) antibodies. The scale bar is 10 μM. (**E**,**F**) The horizontal lines represent the mean ± SEM of five independent experiments. *, **, *** and **** represent *p* < 0.05, *p* < 0.01, *p* < 0.001 and *p* < 0.001, respectively, for Tukey’s multiple comparisons test.

Synaptic plasticity occurs due to LTP induction in which dendritic spine volume has been changed and the synapses strengthened [3,4]. It has been reported that chemical LTP increases the dendritic spine volume through NMDAR-mediated manners. NMDAR antagonists (D-APV and MK-801) decrease the spine size that was increased by chemical LTP in cultured neurons [24]. To examine whether LTP induces the phosphorylation of PSD-93 in primary striatal neurons, we employed a chemically (glycine)-induced LTP method. Along with the continuous release of glutamate from axonal terminals, in this method, glycine can selectively activate synaptic NMDARs while withdrawing Mg^2+^ from the medium using perfusion with glycine containing nACSF buffer [25]. Primary striatal neurons were cultured until the in vitro day (DIV21) and then treated with glycine to induce chemical LTP via NMDAR activation. Chemical LTP induction stimulated the phosphorylation of MYPT1 Thr853 at 10 min (approximately 1.5-fold, ns) and 60 min (approximately 2-fold, *p <* 0.05), an effect that was significantly inhibited (*p <* 0.05 and *p <* 0.01, respectively) by pretreatment of neurons with Y-27632, as described previously [11]. We also found that chemical-LTP induced the phosphorylation of PSD-93 at 10 min (approximately 3-fold, *p <* 0.01) and 60 min (approximately 4-fold, *p <* 0.001), and this phosphorylation was significantly diminished by pretreatment with Y-27632 (*p <* 0.05 and *p <* 0.05, respectively) (Figure 6A–C), indicating that Rho-kinase phosphorylates PSD-93 downstream of NMDARs during LTP induction.

Next, we examined the localization of PSD-93 and PSD-95 in dendritic spines during LTP induction. Primary striatal neurons were cultured for DIV14 and infected with the AAV-CAGGS-Flex-EGFP virus along with the AAV-CaMKII-Cre virus to visualize the dendritic spines. The neurons at DIV21 were pretreated with dimethyl sulfoxide (DMSO) or Y-27632 for 20 min and incubated with glycine for 60 min to induce chemical-LTP. We found that chemical-LTP increased the dendritic spine volume by approximately 50% (*p <* 0.0001), an effect that was significantly suppressed (*p <* 0.001) with Y-27632 pretreatment (Figure 6D,E), as previously described [11]. The colocalization of PSD-93 with PSD-95 increased approximately 30% (*p <* 0.01) after chemical LTP induction in the dendritic spines, but was significantly inhibited by pretreatment with Y-27632 (*p <* 0.05) (Figure 6D,F). Revealing the role of Rho-kinase in colocalization of PSD-93 with PSD-95 in dendritic spines helps to better understand the structural synaptic plasticity in neurons.

## 3. Discussion

RhoA and its effector, Rho-kinase, are considered to be important for synaptic functions, but the mechanism by which Rho-kinase regulates synaptic functions is not yet fully understood. In this study, we examined whether Rho-kinase phosphorylates PSD-93, one of the major postsynaptic regulatory proteins, and found that Rho-kinase indeed phosphorylates PSD-93 downstream of NMDARs. The phosphorylation of PSD-93 appears to increase the interaction and colocalization of PSD-93 with PSD-95 in dendritic spines to regulate structural plasticity.

### 3.1. Phosphorylation of PSD-93

Similar to other MAGUK family proteins, PSD-93 has the potential to interact with other postsynaptic proteins, and this interaction is suggested to be phosphorylation dependent [26]. Several studies have been conducted on the phosphorylation dependent regulation of PSD-93, and one study has shown that extracellular signal-regulated kinases (ERKs) phosphorylate PSD-93 at Ser323 in striatal neurons, but the role of this phosphorylation remains unknown [27]. Another study has demonstrated that Fyn kinase phosphorylates Thr384 of PSD-93 and thus upregulates NMDAR function [28,29]. Other publications have revealed that PSD-93 deletion leads to the mislocalization of Fyn from the synaptosomal membrane. As a result, tyrosine phosphorylation of NR2A and NR2B is depleted [30]. These findings indicate that PSD-93 acts as a membrane-anchored substrate of Fyn and plays a major role in the regulation of Fyn-mediated upregulation of NMDAR function [31]. In the present study, we found that Rho-kinase directly phosphorylated PSD-93 in the PDZ3-SH3 domain in vitro (Figure 1A–C). In contrast, Rho-kinase did not phosphorylate PSD-95 in vitro. We also found that Rho-kinase phosphorylated PSD-93 at four different sites (Thr585, Ser590, Ser598, and Thr612) in vitro (Figure 1D–F). Among these four phosphorylation sites, the major phosphorylation sites were Thr585 and Thr612. Stimulation of striatal slices with an NMDAR agonist induced Rho-kinase-mediated phosphorylation of PSD-93 at Thr612 (Figure 3A–D). Chemical-LTP increased the phosphorylation of PSD-93 at Thr612 in a Rho-kinase-dependent manner (Figure 6A–C). These results indicate that Rho-kinase phosphorylates PSD-93 at Thr612 downstream of NMDARs during synaptic plasticity.

### 3.2. Roles of PSD-93 Phosphorylation in PSD Complex Formation

MAGUK family proteins can bind to AMPARs and NMDARs via their PDZ domains [32]. PSD-93 is a MAGUK family protein that is closely related to PSD-95 in terms of expression, amino acid sequence, domain organization and functions [23]. In this study, PSD-93 was immunoprecipitated with PSD-95, the NR1 subunit of NMDARs, and the GluR1 subunit of AMPARs. Treatment with calyculin A enhanced this effect, whereas pretreatment with Y-27632 inhibited this effect (Figure 5A–D). Ectopic expression of PSD-93 and PSD-95 in COS7 cells resulted in an interaction between PSD-93 and PSD-95. This interaction was facilitated by the phosphorylation of PSD-93 at Thr612 and Thr585 of PSD-93 by Rho-kinase (Figure 5E,F). These results suggest that PSD-93 and PSD-95 form heterodimers in a phosphorylation-dependent manner and that the interaction contributes to LTP induction by stabilizing NMDAR localization at the plasma membrane [14,33]. In contrast, Rho-kinase negatively regulates the interaction of PSD-93 with SynGAP1, ADAM22, and LGI1 (Supplementary Materials, Figure S3A–D). PSD-93 can interact with SynGAP1 via the RasGAP domain (amino acids 670–685) [34]. In our previous study, we found that Rho-kinase phosphorylates SynGAP1 [11]. Thus, Rho-kinase phosphorylates both SynGAP1 and PSD-93, thereby dissociating SynGAP1 from the heterodimer of PSD-93 and PSD-95. The dissociation of SynGAP1 from the PSD region leads to the activation of the Ras–ERK pathway and promotes spine enlargement [11,35]. ADAM22, LGI1, and MAGUKs (including PSD-95 and PSD-93) form protein complexes in vivo and play an important role in synaptic transmission via AMPARs and NMDARs [36]. In the present study, we found that Rho-kinase phosphorylates the PDZ3-SH3 domain of PSD-93, which is the binding region of ADAM22, and negatively regulates the binding of PSD-93 to ADAM22 and LGI1 in a phosphorylation-dependent manner (Supplementary Materials, Figure S3A,C,D). However, the functional role of the phosphorylation-dependent regulation of PSD-93 binding to ADAM22 and LGI1 by Rho-kinase requires further elucidation.

### 3.3. Roles of PSD-93 Phosphorylation in Synaptic Plasticity

NMDAR stimulation induces Ca^2+^ influx into neurons, activates CaMKII and further activates the RhoA-Rho-kinase pathway, leading to dendritic spine enlargement and LTP [7,11] (Supplementary Materials, Figure S3). However, the mechanism by which Rho-kinase regulates spine enlargement and LTP remains poorly understood. To clarify the function of Rho-kinase in synaptic plasticity, we focused on PSD-93 among the Rho-kinase candidate substrates. MAGUK family proteins including PSD-93 directly interact with NMDARs and indirectly interact with AMPARs via stargazin/TARP, contributing to the membrane localization and stabilization of these receptors [37]. In fact, targeted disruption of the PSD-93 gene not only reduces surface NR2A and NR2B expression, but also NMDAR-mediated excitatory postsynaptic currents and potentials [38]. Simultaneous knockdown/knockout of three MAGUK members—PSD-93, PSD-95 and SAP102—reduces almost all AMPAR-mediated synaptic transmission in rat hippocampal slice cultures [23,39,40]. In the present study, we found that PSD-93 phosphorylation by Rho-kinase promoted the interaction of PSD-93 with PSD-95 (Figure 5A,B,E,F). Chemical LTP induction increased dendritic spine volume and the colocalization of PSD-93 with PSD-95 in cultured striatal neurons (Figure 6D,E). On other hand, PSD-93 phosphorylation by Rho-kinase decreased the interaction with SynGAP1 (Supplementary Materials, Figure S3A,B,E,F). The delocalization of SynGAP1 from postsynaptic density region increases dendritic spine size [11]. Taken together, these results suggest that Rho-kinase positively regulates the interaction of PSD-93 with PSD-95, NMDARs and AMPARs, and negatively regulates the interaction of PSD-93 with SynGAP1, ADAM22 and LGI1 for orchestrating synaptic functions as depicted in Supplementary Materials, Figure S3. Nonetheless, further electrophysiological and behavior studies should be done not only with conditional knockout but also total knockout mice to elucidate a clearer understanding.

## 4. Materials and Methods

### 4.1. Animals

Male C57BL/6J (RRID: MSR_JAX:000664) and pregnant female ICR mice (RRID: IMSR_JAX:009122) were purchased from Japan SLC (Hamamatsu, Shizuoka, Japan). The four mice were kept in a cage (17 cm wide × 28 cm long × 13 cm high) in an animal facility that was pathogen free under standard conditions (23 ± 1 °C, 50 ± 5% humidity) and a 12-h light/dark cycle (light phase 9:00–21:00). All mice had free access to food and water. The mice were carefully handled by laboratory personnel in order to reduce their suffering. Between control and experiment, the mice were randomly chosen. All animal experiments were approved and performed in accordance with the guidelines for the care and use of laboratory animals established by the Animal Experiments Committee of Nagoya University Graduate School of Medicine (approval number: 20094) and Fujita Health University (approval number: AP20037). All experiments were conducted in compliance with the ARRIVE guidelines.

### 4.2. Materials

A rabbit polyclonal anti-pT612 PSD-93 antibody was produced against the phos-pho-peptide, C+RARLKpT612VKFN (Merck, Kenilworth, NJ, USA). The phospho-specific antibody was purified with an affinity column with phosphopeptide by applying peptide in a Sulfolink coupling gel (Thermo Fisher Scientific, Waltham, MA, USA). Rabbit polyclonal anti-MBS (MYPT1) antibody was generated by use of GST-rat MBS-N-terminal domain (1-707 aa) [18]. A rabbit anti-ADAM22 R4 antibody was kindly provided by Dr. Masaki Fukata (National Institute for Physiological Sciences, Aichi, Japan) [41]. The following antibodies were obtained commercially: rabbit anti-phospho-MYPT1 (T853) (RRID:AB_310812), mouse anti-GluR1 (RRID: AB_11212678) (EMD Millipore, Billerica, MA, USA), rabbit anti-MBS (MYPT1) (homemade), rabbit anti-PSD-93 (RRID: AB_2039808) (Alomone Labs, Jerusalem, Israel), mouse anti-PSD-95 (RRID:AB_795156) (Thermo Fisher Scientific), rabbit anti-c-Myc (A-14) (RRID: AB_631274) (Santa Cruz, Dallas, TX, USA), mouse anti-EGFP (RRID: AB_390913) (Roche Diagnostics, Basel, Switzerland), rat monoclonal anti-EGFP (RRID:AB_10013361) (Nacalai Tesque Inc., Kyoto, Japan), mouse anti-GST (RRID: AB_2883970) (Fujifilm Wako, Osaka, Japan), rabbit anti-phospho-CaMKII (T286) (RRID:AB_2713889), rabbit anti-CaMKII (RRID:AB_10545451) (Cell Signaling Technology, Inc., Danvers, MA, USA), rabbit anti-SynGAP1(RRID: AB_11141232), (Abcam, Cambridge, UK); mouse anti-NR1 (RRID: AB_10002447) (Novus Biologicals, Littleton, Co., USA), and mouse anti-LGI1 (RRID:AB_2735259) (Thermo Fisher Scientific). The following secondary antibodies were also obtained commercially: donkey polyclonal anti-rat IgG with Alexa Fluor 488 (RRID: AB_2535794), donkey polyclonal anti-rabbit IgG with Alexa Fluor 555 (RRID: AB_162543), goat polyclonal anti-rabbit IgG with Alexa Fluor 680 (RRID: AB_2535758), goat polyclonal anti-mouse IgG with Alexa Fluor 680 (RRID: AB_2535724) (Thermo Fisher Scientific) and goat anti-mouse IgG (H + L DyLight 800 Conjugate) (RRID: AB_10693543) (Cell Signaling Technology). The following reagents were used: Dimethyl sulfoxide (DMSO, Merck), calyculin A (Fujifilm Wako), Y-27632 (Fujifilm Wako), NMDA (Merck), KCl (Merck).

### 4.3. Plasmid Construction

The cDNA encoding mouse PSD-93 and PSD-95 was amplified by PCR (DNA Engine, Peltier Thermal Cycler, BIO-RAD, CA, USA) from mouse cDNA. SynGAP1-encoding cDNA was purchased from the Kazusa DNA Research Institute (Chiba, Japan). The PCR-amplified cDNA (encoding full-length or fragments of PSD-93, PSD95 or SynGAP1) was subcloned into pCR-Blunt II-TOPO vector (Thermo Fisher Scientific). After sequencing, these cDNAs were further subcloned into pCAGGS-myc-KK1, pEF-BOS-GST or pGEX expression vectors. The phospho-deficient mutant of PSD-93-T585A, -S590A, -S598A and -T612A were generated with PrimeSTAR mutagenesis basal kit (Takara) by changing T585, S590, S598 and/or T612 into alanine. GST-tagged PSD-93 proteins were produced in BL21 (DE3) Escherichia coli and purified on glutathione-Sepharose 4B beads (GE Healthcare, Chicago, IL, USA). AAV-CAGGS-Flex-EGFP-P2A-WRPE and AAV-CaMKII-Cre were constructed as previously reported [42].

### 4.4. Striatal Neuronal Culture

Primary striatal neurons were collected and isolated from E15-E16 mouse embryos using papain as previously described [43]. In this method, pregnant ICR mice were sacrificed by cervical dislocation. Then the brains were dissected humanly from the mouse embryos and kept in 1 × HBSS (Thermo Fisher Scientific) solution. Later, the striatal neurons were prepared from the collected embryo striatum using neuron dissociation solutions (FUJIFILM Wako, Tokyo, Japan) according to manufacturer’s protocol. The neurons were seeded on 60 mm dishes that had been previously coated with poly-D-lysine (PDL; Merck). After 2 h of incubation with Neurobasal Medium^TM^ (Thermo Fisher Scientific) along with 10% fetal bovine serum (FBS; Merck), the medium was replaced with Neurobasal Medium^TM^ (Thermo Fisher Scientific) supplemented with B-27 (Thermo Fisher Scientific) and 1 mM GlutaMAX (Thermo Fisher Scientific). The neurons were cultured until DIV21 in a humidified atmosphere with 5% CO_2_ at 37 °C so that they could mature and develop the dendritic spines. After maturation, the neurons were used for experiments.

### 4.5. In Vitro Phosphorylation Assay

An in vitro phosphorylation assay was carried out as previously described [44]. The GST fused PSD-93 domain proteins were produced in *E. coli*. Then the proteins were purified by using glutathione-Sepharose 4B beads (GE Healthcare). The kinase reaction for Rho-kinase were performed in 50µL of a reaction mixture (50 mM Tris/HCl, pH7.5, 5 mM MgCl2, 1 mM EDTA, 1 mM EGTA, 1 mM DTT, 50 μM [γ-32P] ATP [1–20 GBq/mM]), 0.025–0.5 μM Rho-kinase-cat, and 0.75–1 μM purified GST-PSD-93 fragments for 20 min at 30 °C. Then, the reaction mixtures were boiled in SDS sample buffer and subjected to SDS-PAGE and silver staining. The radiolabeled domain proteins were displayed with an image analyzer (FLA9000; GE Healthcare).

### 4.6. Striatal Slice Culture

Samples from 7- to 8-week-old C57BL/6J male mice were used for striatal slice culture. In this method, the mice were sacrificed humanly by beheading. Then the brain of each mouse was immediately collected in Krebs-HCO_3_- buffer (pH 7.4, 124 mM NaCl, 26 mM NaHCO_3_, 10 mM D-glucose, 4 mM KCl, 1.25 mM KH_2_PO_4_ and 1.5 mM CaCl_2_). According to previously described methods [45,46], the mouse brain was coronally sliced (350 μM) using a VT1200S vibratome (Leica Microsystems). Then, the striatal slices were incubated in the Krebs-HCO_3_- buffer containing 10 μg/mL adenosine deaminase (Roche) at 30 °C for 30 min with oxygenation (95% O_2_/5% CO_2_). The adenosine deaminase-containing Krebs-HCO_3_- buffer was changed to fresh Krebs-HCO_3_- buffer, and the slices were incubated at 30 °C for 30 min. The striatal slices were treated with calyculin A (250 nM) for 60 min with or without Y-27632 (20 μM). After drug treatment, the striatal slices were frozen in liquid nitrogen and stored at −80 °C. The proteins were extracted from striatal slices using 1% SDS buffer. A BCA (Fujifil Wako) assay was carried out to quantify the protein concentration in each sample.

### 4.7. Immunoprecipitation Assay

The calyculin A (250 nM for 60 min) and Y-27632 (20 μM for 60 min) treated striatal slices were sonicated immediately with 320 μL of RIPA buffer (pH 7.5, 150 mM NaCl, 50 mM Tris-HCl, 1 mM EDTA, 1% NP-40, 0.1% SDS, 0.5% sodium deoxycholate, protease inhibitor cocktail [Roche], PhosStop [Roche] and calyculin A [50 nM]). The solution was centrifuged at 16,000× *g* at 4 °C for 10 min. The soluble supernatant was transferred to a new tube and incubated with an anti-PSD-93 antibody (1–2 μg) along with a control rabbit anti-IgG antibody (1–2 μg). The samples were gently rotated on a rotator at 4 °C for 1 h. Next, 25 μL of Protein A Sepharose beads (20% ethanol) were added to each tube, and the tubes were rotated for 1 h. The proteins unbound to beads were washed out with a wash buffer (150 mM NaCl, 50 mM Tris-HCl, 1 mM EDTA, pH 7.5). Immunoblotting and LC-MS/MS were then performed on the immunoprecipitated samples.

### 4.8. Mass Spectrometry

The mass spectrometry (MS) was performed as previously described by [45] with some modifications. The mouse striatal slices were treated with calyculin A (250 nM) with or without Y-27632 (20 μM). Subsequently, an immunoprecipitation assay with an anti-PSD-93 antibody was performed as described previously. The PSD-93-interacting proteins were later isolated from the beads by rotating with guanidine solution (7 M guanidine and 50 mM Tris) for 1 h. The disulfide bond of proteins was then reduced with 5 mM dithiothreitol for 30 min. The hydroxyl group of amino acids were alkylated with 10 mM iodoacetamide for 1 h in the dark. A trypsin solution (50 mM NH_4_HCO_3_, 1.2 M urea, and 0.5 μg of Trypsin/Lys-C) was used to make small peptides from proteins. Desalting was performed by using SPEC tips (Nikkyo Technos, Tokyo, Japan) according to the manufacturer’s protocol. The peptides were analyzed by LC-MS using an Orbitrap Fusion Mass Spectrometer (Thermo Fisher Scientific Inc., MA, USA) coupled to an UltiMate 3000 RSLCnano LC system (Dionex Co., Amsterdam, The Netherlands) using a nano HPLC capillary column (150 mm × 75 m id, Nikkyo Technos Co., Tokyo, Japan) via a nanoelectrospray ion source.

Reversed-phase chromatography was performed with a linear gradient (0 min, 5% B; 100 min, 40% B) of solvent A (2% acetonitrile with 0.1% formic acid) and solvent B (95% acetonitrile with 0.1% formic acid) at an estimated flow rate of 300 nl/min. A precursor ion scan was carried out using a 400–1600 mass-to-charge ratio (m/z) prior to tandem MS (MS/MS) analysis. Tandem MS was performed by isolation at 0.8 Th with the quadrupole, HCD fragmentation with a normalized collision energy of 30%, and rapid scan MS analysis in the ion trap. Only those precursors with a charge state of 2–6 were sampled for MS2. The dynamic exclusion duration was set to 10 sec with a 10-ppm tolerance. The instrument was run in top speed mode with 3 sec cycles. A peak list was generated and calibrated using MaxQuant software [47]. Database searches against the reference proteome of *Mus musculus* obtained from UniProtKB were performed using MaxQuant software. False discovery rates (FDRs) at the peptide, protein, and site levels were set to 0.01. The peak ion intensities obtained from two independent experiments were analyzed.

### 4.9. Cell Culture

From a 1 mL stock that was stored at −80 °C, the COS7 (ATCC, Manassas, VA, USA) cells were cultured in 100-mm plates. The cells were cultured overnight at 37 °C with Dulbecco’s modified Eagle’s medium (DMEM) (Merck) containing 10% FBS (Merck) supplements and 5% CO_2_ in a humidified atmosphere. The cells were incubated until they reached 70–80% confluence. Then, the culture plates were randomly chosen for control and experiments. The pEF-BOS-GST and pCAGGS-Myc plasmids were transformed with Lipofectamine 2000 (Thermo Fisher Scientific) into cells according to the manufacturer’s protocols. Later the cells were treated with calyculin A (50 nM for 12 min) with or without Y-27632 (20 μM for 30 min). The stimulated cells were collected with lysis buffer (pH 7.5, 150 mM NaCl, 20 mM Tris-HCl, 1 mM EDTA, 1% NP-40, PhosStop [Roche], protease inhibitor cocktail [Roche] and 50 nM calyculin A) for the GST pull-down assay.

### 4.10. GST Pull-Down Assay

The pEF-BOS-GST and pCAGGS-Myc plasmids were transfected with lipofectamine 2000 (Thermo Fisher Scientific) into COS7 cells according to the manufacturer’s protocols. Next, the COS7 cells were treated with 50 nM calyculin A with or without 20 μM Y-27632 as previously described [48]. The stimulated cells were sonicated with 700 μL of lysis buffer (pH 7.5, 150 mM NaCl, 20 mM Tris-HCl, 1 mM EDTA, 1% NP-40, protease inhibitor cocktail [Roche], and PhosStop [Roche], 50 nM calyculin A). The solution was then centrifuged at 16,000× *g* for 10 min at 4 °C. The supernatant was transferred to a fresh tube and incubated in a rotor with 20 μL of glutathione-Sepharose 4B beads (GE Healthcare) for 1 h at 4 °C. After rotation, the unbound GST-proteins were removed from the beads by washing with wash buffer (pH 7.5, 20 mM Tris-HCl, 1 mM EDTA, 150 mM NaCl). The beads were then boiled for 10 min with a 1× SDS buffer. Finally, the boiled samples were subjected to immunoblotting with the indicated antibodies.

### 4.11. Immunoblotting

Protein (5–20 μg) from each sample was subjected to SDS-PAGE on 7–8% acrylamide gels. The individual proteins were separated using SDS-PAGE. After SDS-PAGE, the proteins were transferred from the gel onto polyvinylidene difluoride membranes (Immobilon-FL, Millipore, Bedford, MA, USA) using a Trans-Blot Turbo system. Next, the Blocking One buffer (Nacalai Tesque, Kyoto, Japan) was used to block the membranes at room temperature for 30 min. Depending on the primary antibody, the membranes were incubated overnight at 4 °C or at room temperature for 1 h. Anti-PSD-93 (1:1000), anti-phospho-PSD-93 (T612) (1:100), anti-PSD-95 (1:1000), anti-phospho-MYPT1 (T853) (1:1000), anti-MYPT1 (1:1000), anti-c-Myc (1:1000), anti-EGFP (1:1000), anti-GST (1:1000), anti-phospho-CaMKII (T286) (1:1000), anti-CaMKII (1:1000), anti-SynGAP1(1:1000), anti-GluR1 (1:1000), anti-NMDAR1 (1:1000), anti-ADAM22 (1:1000), and anti-LGI1 (1:1000) were used. The unbound primary antibodies were washed away, and the membranes were incubated with goat anti-rabbit Alexa Fluor 680 and/or goat anti-mouse IRDye 800CW for 1 h at room temperature. The total protein or phospho-proteins were detected by infrared imaging (LI-COR Biosciences Lincoln, NE). The band intensities were quantified using ImageStudio software (LI-COR Biosciences).

### 4.12. Chemical LTP Assay

Primary striatal neurons were dissected from E16 embryonic mice and cultured according to the protocol described in striatal neuronal culture. After DIV21, the neurons were used for chemical LTP induction as previously described [11]. In this method, the neurons were treated with Y-27632, then nACSF was added (125 mM NaCl, 2 mM CaCl_2_, 2.5 mM KCl, 1 mM MgCl_2_, 33 mM glucose, 5 mM HEPES, 0.5 μM TTX, 20 μM bicuculline and 3 μM strychnine; pH 7.3), and the neurons were incubated for 10 min. Next, the medium was changed to 200 μM glycine containing nACSF (pH 7.3, 125 mM NaCl, 2 mM CaCl_2_, 2.5 mM KCl, 5 mM HEPES, 33 mM glucose, 0.5 μM TTX, 20 μM bicuculline and 3 μM strychnine) and incubated for 5 min. Finally, the neurons were incubated with the original Neurobasal Medium^TM^ for 10 min and 60 min. The neurons were collected with 1xSDS and boiled for 10 min. Western blotting was performed on the boiled samples to examine PSD-93 phosphorylation.

### 4.13. AAV Virus Preparation and Transfection

Three plasmids, an AAV vector, pAAV-DJ (Cell Biolabs, San Diego, CA, USA) and pHelper were transfected into 80–90% confluent AAV293 cells (Cell Biolabs). After 3 days of incubation, cells were harvested. AAV vectors produced in AAV293 cells were purified by CsCl (Nacalai Tesque) gradient ultracentrifugation using a SW41Ti rotor (Beckman-Coulter Life Science, Indianapolis, IN) at 35,000 rpm for 72 h at 16 °C. The formed gradients were fractionated, and the AAV titer of each fraction was evaluated by qPCR (TOYOBO, Osaka, Japan). AAV-rich fractions were combined and dialyzed using a dialysis cassette Slide-A-Lyzer (Thermo Fisher Scientific) to remove CsCl. The final AAV titer was determined by qPCR.

### 4.14. Immunostaining

Neurons were fixed with 4% paraformaldehyde (PFA) in phosphate-buffered saline (PBS) for 10 min at RT. Neurons were treated with 0.05% Triton X-100/PBS for 10 min on ice and blocked with 10% donkey serum albumin in PBS. Neurons were incubated with Rat anti-GFP (1:1000 dilution), Rabbit anti-PSD-93 (1:100 dilution) and Mouse anti-PSD-95 (1:100 dilution) antibodies for 1 h at RT. After washing with PBS for 10 min repeated three times, the neurons were incubated with Rat Alexa Fluor 488-, Rabbit Alexa Fluor 555- or Mouse Alexa Fluor 647-conjugated secondary antibodies for 1 h and unbound anti-bodies were washed away with PSB for 10 min repeated three times. Confocal images were recorded with LSM 780 microscopes built around an Axio Observer Z1 with Plan-Apochromat 20× (numerical aperture [NA] 0.8) or Plan-Apochromat 63× (NA 1.40) lenses under the control of ZEN Digital Imaging for Light Microscopy (RRID: SCR_013672, Carl Zeiss, Oberkochen, Germany). The Imaris software version 9.8.2 (Bitplane, Oxford Instruments, Belfast, UK) was used to draw 2D dendritic spines from the captured LSM-780 images. Furthermore, this software was used for the spine volume quantification and colocalization of the PSD-93 and PSD-95 proteins in the dendritic spines of the cultured neurons.

### 4.15. Statistical Analysis

The samples were chosen randomly without using any randomization methods. The mean ± SEM of the data were calculated using at least three different independent experiments. The statistical analysis was performed with Prism version 6 software (GraphPad, San Diego, CA, USA) using one-way ANOVA test, followed by Tukey’s or Dunnett’s multiple comparison test. Differences between two samples were considered significant if the *p* value was <0.05 (* *p <* 0.05; ** *p <* 0.01; *** *p <* 0.001; and ****p < 0.0001).

## Figures and Tables

**Table 1:** Phosphorylation-dependent positively regulated PSD-93 binding partners. LC-MS/MS data were sorted according to two criteria: 1. the sample-control vs. IgG-control ratio was 10:1; and 2. binding with PSD-93 was greater upon calyculin A stimulation than under the sample-control condition or Y-27632 stimulation. The list shows the proteins that were positively regulated upon the phosphorylation of PSD-93 by Rho-kinase.

List of Phosphorylation Dependent Positively Regulated PSD-93 Binding Partners						
Agap3	Dlg1	Dlgap1	Dlg4	Dlgap4	Grin1	Grin2a
Grin2b	Grik5	Iqsec2	Nrcam	Rtn3		

**Table 2:** Phosphorylation dependent negatively regulated PSD-93 binding partners. LC-MS/MS data were sorted according to two criteria: 1. the sample-control vs. IgG-control ratio was greater than 10:1, and 2. binding with PSD-93 upon calyculin A stimulation was lower than that under the sample-control condition or after Y-27632 stimulation. The proteins in the box were negatively regulated upon PSD-93 phosphorylation by Rho-kinase.

List of Phosphorylation Dependent Negatively Regulated PSD-93 Binding Partners						
ADAM22	Begain	Cacna2d1	Cit	Cnksr2	Dclk1	Dlgap2
Dlgap3	Griks	Kcnj4	Lrrc7	LGI1	Map4	Prph
Shank3	Shisa7	SynGAP1				

## Data Availability

The data presented in this study are available in https://kanphos.neuroinf.jp (accessed on 10 December 2021).

## Supplementary Materials

The supporting information can be downloaded at: https://www.mdpi.com/article/10.3390/ijms24010404/s1.
