# Supplementary material for: Rho-Kinase/ROCK Phosphorylates PSD-93 Downstream of NMDARs to Orchestrate Synaptic Plasticity
Source: Int J Mol Sci. 2022 Dec 26;24(1):404. doi: 10.3390/ijms24010404 (PMC9820267; doi:10.3390/ijms24010404)
Supplement: Supplementary file 1 [file ijms-24-00404-s001.zip › ijms-2073948-supplementary.pdf]

Rho-Kinase/ROCK Phosphorylates PSD-93 Downstream of NMDARs to Orchestrate Synaptic Plasticity

Emran Hossen [1,2], Yasuhiro Funahashi [2], Md. Omar Faruk [1,3], Rijwan Uddin Ahammad [1,4], Mutsuki Amano [1], Kiyofumi Yamada [5] and Kozo Kaibuchi [1,2,*]

[1] Department of Cell Pharmacology, Graduate School of Medicine, Nagoya University, 65 Tsurumai, Nagoya 466-8550, Japan
[2] Division of Cell Biology, International Center for Brain Science, Fujita Health University, Toyoake 470-1192, Japan
[3] Department of Biochemistry and Molecular Biology, Faculty of Biological Sciences, University of Dhaka, Dhaka 1000, Bangladesh
[4] Department of Cellular and Molecular Medicine, University of California San Diego, La Jolla, CA 92093, USA
[5] Department of Neuropsychopharmacology and Hospital Pharmacy, Graduate School of Medicine, Nagoya University, 65 Tsurumai, Nagoya 466-8550, Japan
* Correspondence: kaibuchi@fujita-hu.ac.jp; Tel.: +81-0562-93-2683

Supplemental Materials: The following supporting information can be downloaded at: www.mdpi.com/xxx/s1, Figure S1: Validation of the phospho-specific PSD-93 Thr612 antibody, related to Figure 3.; Figure S2: PSD-93 phosphorylation negatively regulates the interaction of PSD-93 with SynGAP1, ADAM22 and LGI1, related to Figure 5.; Figure S3: Graphical abstract showing the role of PSD-93 phosphorylation induced by Rho-kinase in synaptic orchestration.

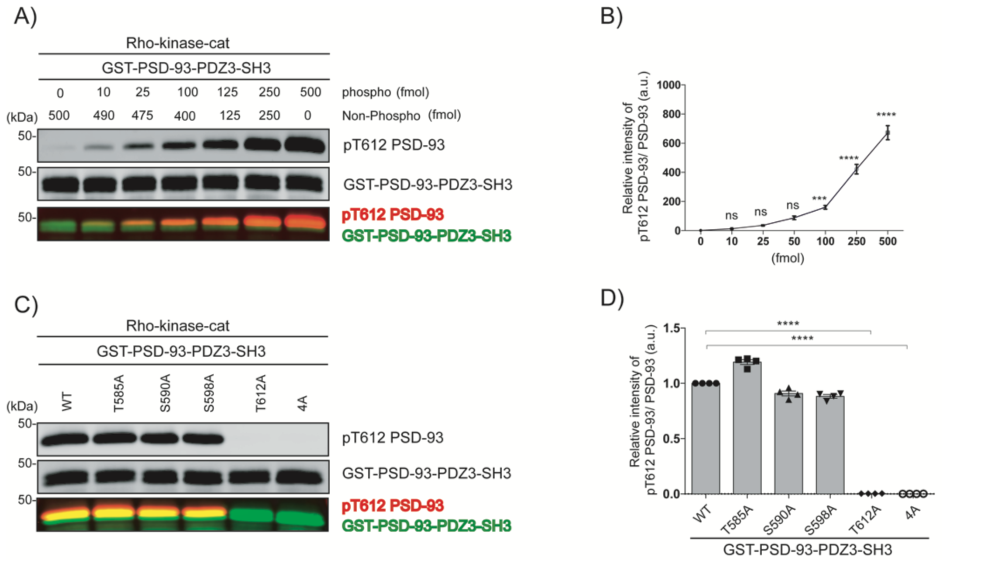

Supplemental Figure S1: Validation of the phospho-specific pT612 PSD-93 antibody, related to Figure 3. A) A phospho-specific antibody against T612 phosphorylation detected pT612 PSD-93 phosphorylation in a dose dependent manner. The left panel shows the indicated amounts of phosphorylated and nonphosphorylated PSD-93 PDZ3-SH3 proteins that were mixed and subjected to immunoblot analysis with anti-pT612 PSD-93 and GST antibodies. The double-color image represents phospho-protein (red) and total protein (green). B) The right panel shows the quantification of the immunoblotting data of pT612 PSD-93. The horizontal lines indicate the mean ± SEM of four independent experiments. *** and **** represent $p < 0.001$, $p < 0.0001$, respectively and "ns" denotes "not significant", Dunnett's multiple comparisons test. C) Specificity of the anti-pT612 PSD-93 antibody. Bottom left panel shows the immunoblot data after incubation of PSD-93 PDZ3-SH3 WT and PSD-93 T612A PDZ3-SH3 protein with [γ^{32}P] ATP in the presence of constitutively active Rho-kinase, the samples were analyzed with anti-pT612 PSD-93 and anti-GST antibodies. The double-color image

represents phospho-protein (red) and total protein (green). D) The right panel shows the quantification of the immunoblotting data obtained using the anti-pT612 antibody. The horizontal lines indicate mean ± SEM of four independent experiments. **** represents $p < 0.0001$, Dunnett's multiple comparisons test.

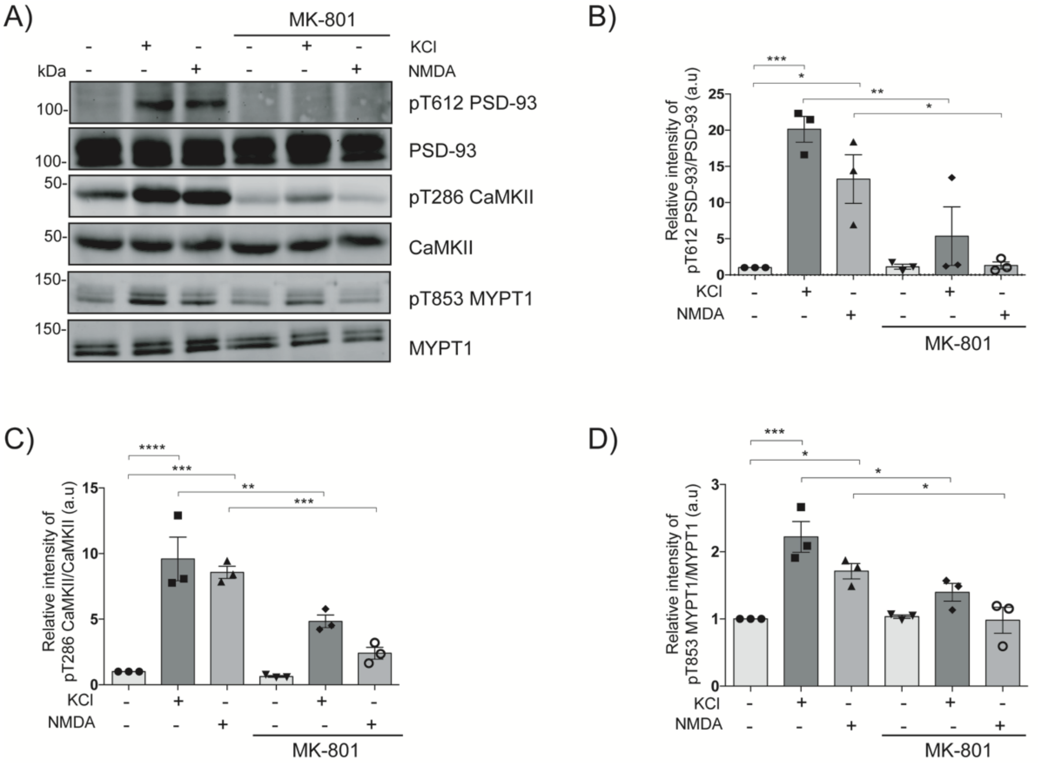

Supplemental Figure S2: Rho-kinase phosphorylates PSD-93 at Thr612 downstream of NMDAR-CaMKII cascade in striatal slices, related to figure 3. A) Rho-kinase phosphorylates PSD-93 through NMDARs stimulation. Striatal slices were treated with or without MK-801 (100 μM) for 60 min and then treated with high potassium (KCl 40 mM) for 15 sec or NMDA (100 μM) for 15 sec. B) C) D) The quantification of immunoblotting data of pT612 PSD-93, pT286 CaMKII, and pT853 MYPT1. The horizontal lines indicate mean ± SEM of three independent experiments. *, **, *** and **** represent $p < 0.05$, $p < 0.01$, $p < 0.001$ and $p < 0.0001$, respectively, Tukey's multiple comparisons test.

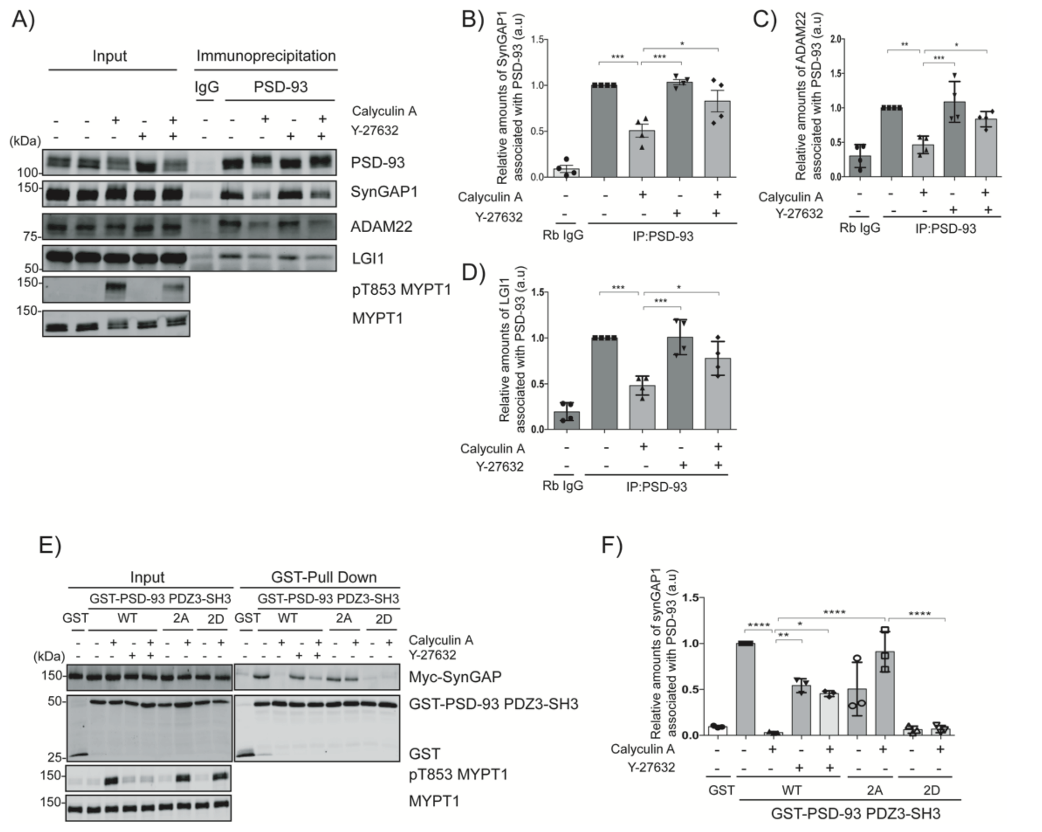

Supplemental Figure S3: Rho-kinase negatively regulates the interaction of PSD-93 with SynGAP1, ADAM22 and LGI1, related to Figure 4. A) PSD-93 phosphorylation by Rho-kinase decreases its interaction with SynGAP1, ADAM22 and LGI1 in striatal slices. Striatal slices were treated with calyculin A (250 nM) for 60 min after they were pretreated with Y-27632 (20 μM) for 60 min and subjected to immunoprecipitation using an anti-PSD-93 antibody. The precipitated proteins were subjected to immunoblot analysis with anti-PSD-93, SynGAP1, ADAM22 and LGI1 antibodies. B) C) D) The right panel shows the statistical analysis of the immunoblot data. The horizontal lines indicate the mean ± SEM of four independent experiments. *, ** and *** represent $p < 0.05$, $p < 0.01$ and $p < 0.001$ respectively, Tukey's multiple comparisons test. E) PSD-93 phosphorylation by Rho-kinase decreases the interaction between PSD-93 and SynGAP1 in COS7 cells. COS7 cells were transfected with the indicated plasmids, and then treated with calyculin A (50 nM) for 12 min after they were pretreated with Y-27632 (20 μM) and then subjected to GST pull down assay. The left panel shows the GST-PSD-93-PDZ3-SH3, Myc-SynGAP1, pT853 MYPT1, and MYPT1. F) The right panel shows the quantification of the immunoblot data. The horizontal lines indicate the mean ± SEM of three independent experiments. *, ** and **** represent $p < 0.05$, $p < 0.01$ and $p < 0.0001$, respectively, Tukey's multiple comparisons test.
